# Isoliensinine Suppresses Osteoclast Formation Through NF-κB Signaling Pathways and Relieves Ovariectomy-Induced Bone Loss

**DOI:** 10.3389/fphar.2022.870553

**Published:** 2022-07-22

**Authors:** Huijiang Liu, Ronghe Gu, Qian Huang, Yun Liu, Chong Liu, Shijie Liao, Wenyu Feng, Tianyu Xie, Jinmin Zhao, Jiake Xu, Qian Liu, Xinli Zhan

**Affiliations:** ^1^ Department of Orthopedics, The First Affiliated Hospital of Guangxi Medical University, Nanning, China; ^2^ Department of Orthopedics, The First People’s Hospital of Nanning, Nanning, China; ^3^ Guangxi Key Laboratory of Regenerative Medicine, Orthopedic Department, The First Affiliated Hospital of Guangxi Medical University, Nanning, China; ^4^ School of Biomedical Sciences, University of Western Australia, Perth, WA, Australia

**Keywords:** isoliensinine, osteoporosis, NF-κB pathway, osteoclast, receptor activator of nuclear factor-κB ligand (RANKL)

## Abstract

Osteoporosis is among the major contributors of pathologic fracture in postmenopausal women, which is caused by the bone metabolic disorder owing to the over-activation of osteoclasts. Inhibition of osteoclast differentiation and maturation has become a mainstream research interest in the prevention of osteoporosis. Isoliensinine (Iso) is a dibenzyl isoquinoline alkaloid with antioxidant, anti-inflammatory, and anti-cancer activities. However, whether it can be used as a potential treatment for osteoporosis remains undiscovered. Here, we investigated whether Iso might suppress the differentiation of osteoclasts *in vitro* and *in vivo* to play an anti-osteoporosis role. Our results showed that Iso inhibits the formation of mature multinuclear osteoclasts induced by RANKL, the bone resorption, and the osteoclast-specific genes expression by blocking the nuclear translocation of NF-κB p65, and the effect was in a dosage-dependent way. Furthermore, we investigated the therapeutic effect of Iso on osteoporosis in ovariectomized (OVX) mice. We found that Iso attenuated bone loss in the OVX mice and significantly promoted BS, Conn. DN, Tb.Th, TB.N, and BV/TV Index. All in all, Iso showed a prominent effect of osteoclast inhibition, with great promise for treating osteoporosis.

## Introduction

Osteoporosis is a systemic illness especially in postmenopausal women whose characteristics include reduced bone mass and damage of bone microstructure, resulting in a pathological fracture in the middle-aged and old crowd, greatly increased health-care costs, orthopedic complications morbidity, and mortality (Compston et al., 2019). Osteoporosis arises on account of an imbalance of bone homeostasis, in which osteoblast-mediated osteogenesis fails adequately to fill the osteoclast-mediated resorption pits (Yang et al., 2020). Even though significant progress has been made in search of efficacious drugs for osteoporosis in nearly 2 decades. Due to concerns about the side-effects of anti-bone resorption drugs (especially bisphosphonates) and the lack of explicit evidence for their long-term efficacy, numerous patients who should benefit from drug treatment do not get the desired effect (Khosla and Hofbauer, 2017). Therefore, it is necessary to develop new drugs with fewer side effects and long-term efficacy to improve patients’ compliance and obtain better curative effects.

Mature osteoclasts are derived from osteoclast precursors which gradually differentiated from hematopoietic progenitor cells from bone marrow. The strategy of restraining bone resorption via inhibiting osteoclast differentiation has been accepted by a majority of scholars (Boyce, 2013; Li Q. et al., 2020; Wang et al., 2020). RANKL (Receptor Activator of Nuclear Factor-κ B Ligand), which is mainly derived from osteoblasts, binds to the RANK on the osteoclast membrane and triggers the differentiation and maturation of osteoclast, as well as nuclear genetic programs that activate intracellular kinase cascades and essential transcription factors, including NFATc1 (nuclear factor-activated T cell 1) and c-Fos, and ultimately induce osteoclast differentiation (Edwards and Mundy, 2011). MAPK and NF-κB perform an integral function during the course of osteoclast differentiation (Ono and Nakashima, 2018). Activated NF-κB and MAPK dimers induce activator protein-1 (AP-1). After RANKL stimulation, JDP2, ATF4, NFATc2, AP-1, and NF-ΚB, were recruited in the promoter region of the NFATc1 gene to conduce the expression of NFATc1 (Matsumoto et al., 2004; Asagiri et al., 2005; Cao et al., 2010; Maruyama et al., 2012). NFATc1 is a major transcription factor that is involved in modulating osteoclast differentiation, which drives osteoclast-specific genes, including DCSTAM, ATP6V0D2, Oscar, ITGB3, OCSTAM, ACP5(TRAP), Calcl, and CTSK to stimulate mature osteoclast formation and mediate bone resorption (Matsumoto et al., 2004; Matsuo et al., 2004; Kim et al., 2008; Miyamoto et al., 2012).

Isoliensinine (Iso) is a dibenzyl isoquinoline alkaloid isolated from lotus seed embryos with antioxidant, anti-inflammatory, and anti-cancer activities (Cheng et al., 2021). Research evidence has shown that Iso may stimulate the apoptosis of hepatoma cells by suppressing the activity of NF-κB as well as the p65 and phosphorylation (Shu et al., 2015). However, the role of Iso in osteoclasts and osteoporosis remains unknown. In the present research, we discovered that Iso suppressed the bone resorption, the formation of RANKL-induced osteoclast, and the osteoclast-specific related gene expression. Of interest to us is that Iso inhibits osteoclasts primarily through NF-κB signaling pathways rather than MAPK pathways. Furthermore, the *in vivo* validation of Iso in an ovariectomized mouse model clearly demonstrated the favorable function of Iso in the prevention of bone loss caused by estrogen deficiency. Aforesaid convincing evidence suggests that Iso has a strong protective impact on osteoporosis and osteolytic illnesses.

## Materials and Methods

### Reagents and Materials

Iso was procured from Wuhan ChemFaces Biochemical Co., Ltd. (Hubei, Wuhan, China, #CAS 6817–41–0), and its purity was over 98%. Then, dimethyl sulfoxide (DMSO) was utilized to dissolve it to produce a stock concentration of 100 mmol/L, followed by additional dilution to attain the preferred working concentration utilizing Alpha-modified minimal essential medium (α-MEM). The Thermo Fisher Scientific (Thermo Fisher Scientific, Waltham, MA, United States) provided the fetal bovine serum (FBS) and α-MEM utilized in the present research. PeproTech (Princeton, NJ, United States) supplied the M-CSF (#315–02–10) and RANKL (#315–11–10) whereas Sigma-Aldrich (St. Louis, MO, United States) supplied the tartrate-resistant acid phosphatase (TRAP) staining kits as well as the Cell Counting Kit-8 (CCK-8) assay. Cell Signaling (Danvers, MA, United States United States) supplied all the primary as well as the secondary antibodies used in the present research.

### Network Pharmacology Analysis

We retrieved the 3D structural formula of the Iso drug through the PubChem database (https://pubchem.ncbi.nlm.nih.gov/compound/5274591), after obtaining the structural formula, we logged onto the website (http://www.swisstargetprediction.ch/) to predict Iso target proteins. Then we logged on to the website (https://www.disgenet.org/search) to search for target genes related to osteoporosis diseases. Subsequently, the predicted targets were imported into the Venny 2.1.0 platform together to make the Venn intersection graph. ClusterProfiler (version 3.14.3) was used for KEGG analysis, and visualization was performed through ImageGP online platform.

### Cell Culture and Osteoclastogenesis *in Vitro*

All animal protocols were conducted according to the guidelines stipulated by the Animal Ethics Committee of Guangxi Medical University. C57BL/6J mice aged 6 weeks were procured from the Animal Experiment Center of Guangxi Medical University for experiments. Sacrificing of the mice was performed by cervical dislocation. After disinfection, the tibia and femur were taken from the mice under sterile conditions and placed in a petri dish containing a culture medium. The epiphysis at both ends was removed, followed by additional washing of the marrow cavity with a 1 ml syringe to absorb culture medium until the bone cortex turned white. After being filtrated and centrifuged, the cell suspension was introduced into a T75 culture flask that contained α-MEM in combination with 10 percent FBS, 25 ng/ml M-CSF (complete medium), 100 μg/ml streptomycin, and 100 U/ml penicillin. Subsequently, incubation of the cells was carried out at a temperature of 37°C in an environment containing 95 percent air and 5 percent CO2. On day 3, the culture medium was changed with fresh medium one time, and cells in attachement were utilized in subsequent procedures.

For the purpose of mature osteoclasts generation, BMMs were implanted into a 96-well culture plate with 6×10^3^ cells per well where they were allowed to attach to the culture plates overnight. In the day that followed, BMMs were subjected to stimulation using 50 ng/ml RANKL and 25 ng/mL M-CSF in the presence or absence of increasing concentrations of Iso (0,0.625, 1.25, 2.5 or 5 μM), and replenishing of the culture medium was performed after every 2 days in a duration of over 6 days till the formation of osteoclasts. To thoroughly examine which stage of osteoclast differentiation was affected by Iso, the cells were subjected to treatment using 5 μM Iso on day 1, day 3, and day 5 while being stimulated by RANKL. The positive control consisted of cells that did not receive Iso treatment under stimulation by RANKL. Next, 4 percent paraformaldehyde (PFA) buffer was utilized to fix the cells for 40 min, followed by staining utilizing TRAP reagent to allow for visualization of enzymatic activity. Images of the stained cells were captured utilizing Cytation 5 (BioTek Instruments Inc., Winooski, VT, United States). Cells that were discovered as having a minimum of 3 nuclei were determined to be the osteoclasts and were evaluated utilizing ImageJ software (NIH, Bethesda, MD, United States).

### Cytotoxicity Assay

Seeding of the BMMs was performed in 96-well plates based on a density of 6×10^3^ cells per well at a temperature of 37°C and a concentration of 5 percent CO_2_, then, they were allowed to incubate overnight in order to adhere to the plate walls, followed by treatment with a complete α -MEM medium containing 25 ng/mL M-CSF and increasing dosages of Iso (0,0.625, 1.25, 2.5 or 5 μM) to stimulate the cells for 48 h. Subsequently, the cells were subjected to another incubation for 2 h with CCK-8 solution (10 µl/well). Finally, a TriStar2 LB 942 Multimode Microplate Reader (Berthold Technologies Gmbh & Co. KG, Baden-Württemberg, Germany) was utilized to analyze the absorbance value (OD) at 450 nm.

### Osteoclast Fusion Assay

To further explore the osteoclastogenesis-inhibited effect, the osteoclast fusion assay was performed as previously described (Li X. et al., 2020). In brief, BMMs culture was performed as described above. After 3 days of culture, the adherent cells were washed with PBS for 3 times and implanted into a 96-well culture plate with 6 × 10^3^ cells per well. In the day that followed, BMMs were subjected to stimulation using 50 ng/ml RANKL and 25 ng/mL M-CSF in the presence or absence of increasing concentrations of Iso (0, 2.5 or 5 μM) for 3 days. The cells were next labeled with blue fluorescent nuclear dye Hoechst (H3569, Invitrogen) or red fluorescent cell membrane dye Dil (C7000, Invitrogen) and incubated at room temperature for 10 min. The two groups of cells were cultured on the cell culture plate for 2 h, the culture medium was discarded and observed by fluorescence microscope. The membrane merge rate was quantified using the ImageJ software (NIH, Bethesda, MD, United States).

### Podosomal Actin Belt Staining

To examine the possible impact of actin cytoskeleton on osteoclasts, we induced BMMs via RANKL stimulation in the presence or absence of increasing dosages of Iso (0, 2.5, 5 μM). Following 6 days of culture, fixing of the cells was performed for 30 min utilizing 4 percent PFA, followed by rinsing of the cells thrice using a suitable quantity of PBS. Subsequently, the cells were subjected to incubation for 5 min using 0.1 percent Triton X-100, followed by sealing using 3 percent BSA present in PBS for 30 min. Then, the samples were placed into a dark environment where they were stained utilizing Rhodamine-conjugated phalloidin in 0.2 percent BSA-PBS for 1 h. Then, we rinsed the cells utilizing a suitable proportion of PBS before DAPI staining for 5 min and another washing by PBS. A Cytation 5 was utilized to capture fluorescence images, which were later examined utilizing an automated microscope named BioTek Inc. Instruments (Winooski, VT, United States).

### Bone Resorption Experiment

To determine whether Iso affects the function of osteoclasts, a hydroxyapatite absorption test was carried out as described in the literature (Zhou et al., 2016; Song et al., 2018; Chen et al., 2019). After digesting the BMMs, they were implanted on a 6-well plate having a density of 1 × 10^5^ cells/well. Once the cells had adhered to the bottom overnight, they were stimulated with 50 ng/ml RANKL for 2–3 days till the start of the small osteoclasts formation process. Subsequently, the cells were collected and inoculated on the hydroxyapatite-coated plate (Corning Inc., Corning, NY, United States) at an identical density overnight. Cells were then treated with different concentrations of Iso (0, 2.5, 5 μM) in complete α-MEM containing RANKL and M-CSF. Once 48 h had elapsed, half of the holes in each group were stained using TRAP to calculate the number of osteoclasts. Sodium hypochlorite was utilized to remove the cells on the hydroxyapatite plate in the remaining wells, and the cell resorption area was observed under an optical microscope. Under each experimental condition, the bone resorption area was quantified utilizing ImageJ software (NIH, Bethesda, MD, United States).

### RNA Obtainment and Real-Time Quantitative PCR

qPCR analysis was utilized to examine the expression of osteoclast marker genes. The BMMs were inoculated in 6-well plates (1 × 10^5^cells/well) before stimulation with M-CSF and RANKL in the presence of increasing dosages of Iso (0, 2.5, 5 µM) for 6 days until osteoclasts formed. The TRIzol reagent (Thermo Fisher Scientific) was utilized to isolate total RNA from the cells before the reverse transcription into cDNA using Applied Biosystems ProFlex Base (Thermo Fisher Scientific, Shanghai, China). The following lists the qPCR conditions: denaturation for 10 min at a temperature of 95 °C with 55 cycles, and subsequent reduction of the temperature to 60°C for 15 s before being raised once again to 72°C for 40 s. Table 1 provides a list of the primer sequences utilized in the present research. The analyses were conducted utilizing the 2^−ΔΔCt^ method, followed by the normalization of the target gene expressions to Gapdh.

**TABLE 1:** Primers for qRT-PCR analysis.

Gene	Primer	Sequence (5ʹ-3ʹ)
*c-fos*	Forward	TAC​TAC​CAT​TCC​CCA​GCC​GA
	Reverse	GCT​GTC​ACC​GTG​GGG​ATA​AA
*Mmp9*	Forward	GAA​GGC​AAA​CCC​TGT​GTG​TT
	Reverse	AGA​GTA​CTG​CTT​GCC​CAG​GA
*Acp5*	Forward	ACG​GCT​ACT​TGC​GGT​TTC​A
	Reverse	TCC​TTG​GGA​GGC​TGG​TCT​T
*Ctsk*	Forward	AGG​CGG​CTA​TAT​GAC​CAC​TG
	Reverse	TCT​TCA​GGG​CTT​TCT​CGT​TC
*Nfatc1*	Forward	GGT​GCT​GTC​TGG​CCA​TAA​CT
	Reverse	GAA​ACG​CTG​GTA​CTG​GCT​TC
*Dcstamp*	Forward	TCT​GCT​GTA​TCG​GCT​CAT​CTC
	Reverse	ACTCCTTGGGTTCC TTGCTT
GAPDH	Forward	AAC​TTT​GGC​ATT​GTG​GAA​GG
	Reverse	ACA​CAT​TGG​GGG​TAG​GAA​CA

### Western Blot Assay

To examine the functions of Iso in the process of initial RANKL-mediated signaling events, the BMMs were inoculated at a density of 1 × 10^6^/well into 6-well plates overnight, followed by the incubation of the cells in a FBS-free medium to enable incubation for 3 h before the addition of 2.5 μM Iso for 1 h and cultured again using 50 ng/ml RANKL for 5 min, 10 min, 20 min, 30 min, and 60 min. For long-acting analyses of RANKL, BMMs were inoculated into 6-well plates at 1.5 × 10^5^/well in a complete medium containing 25 ng/ml M-CSF and 50 ng/ml RANKL, in the case where Iso (5 µM) was present or absent for 0, day, 1 day, 3 day, and 5 day.

Lysis of the cells on ice for 30 min was performed utilizing RIPA lysis buffer that contained protease inhibitor and phosphoric acid to acquire the total protein. Then, the loading buffer was introduced into the protein sample and subsequently heated for 15 min at a temperature of 100°C. Once the total protein had been successfully separated utilizing the SDS-PAGE procedure, they were transferred to nitrocellulose membranes (Thermo Fisher Scientific, Shanghai, China), followed by the blocking of the nonspecific immunoreactivities utilizing 5 percent skimmed milk for 1 h at ambient temperature, washing thrice using TBST, and incubation with primary antibody for 14–16 h in a 4° shaker. On the day that followed, the membranes were rinsed using TBST, before incubation for 1 h at ambient temperature with a secondary antibody. Finally, the ImageQuant LAS-4000 system (GE Healthcare, Chicago, Illinois, United States) was utilized to capture the images, which were then subjected to examination utilizing the ImageJ software.

### Molecular Docking

Our previous findings reveal that Iso mainly performs the suppressive function during the early stage of osteoclast differentiation, while MAPK and NF-κB signaling pathways are two indispensable classical pathways in the above differentiation process. To explore the inhibitory mechanism of Iso on osteoclasts, we carried out molecular docking analysis to investigate the potential targets of MAPK and NF-κB signaling pathways that can be bound by Iso. The 3D structures of Iso were obtained from the PubChem website (https://pubchem.ncbi.nlm.nih.gov/), and the 3D structures of target proteins were downloaded from the RCSBPDB database (https://www.rcsb.org/). Based on AutoDock (version 4.2.6) the binding energy and hydrogen bond position of ISO and target proteins were obtained. Finally, the visual output is carried out by PyMol (version 2.0).

### Immunofluorescence p65 Assay

To probe into the potential impacts of Iso on the p65 nuclear translocation, we conducted immunofluorescence staining as reported (Nich et al., 2011). First, seeding of the BMMs (5 × 10^4^ cells/well) was performed in Confocal Dish, followed by incubation for 24 h in a basal medium. Once 3 h of starvation in the serum-free medium had elapsed, Iso was introduced to the sample before the incubation for 1 h, the addition of 50 ng/ml RANKL, and the culturing of cells for 30 min. Subsequently, 4 percent PFA was utilized to fix the cells at room temperature for 20 min, followed by washing thrice using PBS, and incubating for 5 min using 0.1 percent Triton. Then the cells were washed twice using 0.2% BSA-PBS. Diluted P65 in 0.1% BSA-PBS (Dilution 1:300) and the cells were subjected to incubation at 4°C overnight with anti-p65 antibodies. Once the cells had been rinsed thrice using 0.1 percent BSA-PBS and PBS, they were subjected to another incubation at ambient temperature for 45 min with red fluorescent-labeled secondary antibodies, followed by counterstaining of the nuclei in darkness for 5 min using DAPI. Photographs were taken under a confocal microscope (Leica Microsystems CMS GmbH, STELLARIS5, German).

### Ovariectomy Murine Model Establishment

Subsequently, we evaluated the potential impact of Iso on bone loss via the establishment of an osteoporosis model in mice (OVX mice). The Animal Care Committee of Guangxi Medical University granted its approval to all the experiment protocols involving animals. C57BL/6J mice (female, 10 weeks old, 18 ± 0.50 g) were procured from Changsha Tianqin Biotechnology Co., LTD. (Changsha Hubei), and raised in the Animal Center of Guangxi Medical University. After being rested for 1 week, the mice began to be modeled after getting accustomed to the environment. The animal models were categorized into three different groups, namely the OVX group (n = 8), the OVX + Iso group (5 mg/kg *n* = 8), and the sham group (*n* = 8). Anaesthetization of the mice was performed with tribromoethanol, followed by bilateral ovariectomy (in the OVX groups) or sham operation (sham group), in which only the fat near the ovaries is removed. A week later following surgery, 5 mg/kg Iso was administered as an intraperitoneal injection to the mice in OVX + Iso group, whereas the OVX group and Sham group received a control treatment consisting of an intraperitoneal injection of PBS dissolved in 1% DMSO for 42 days. Sacrificing of all the mice was performed through cervical dislocation, followed by the extraction of femurs and the removal of excessive tissue to allow for histological assessment, computed tomography (micro-CT), and microscopic examination.

### Micro-CT Data Analysis

After the sacrifice of mice, the femurs were collected and fixed in 4% PFA for 24 h before the removal of the excessive soft tissues. After 24 h, the 4% PFA was removed, and PBS was utilized to wash the femur twice. Subsequently, the femur was placed in a 1.5  ml EP tube containing 75% alcohol and stored at room temperature. After that, it was sent to Nanchang Shuangjing Biotechnology Co., LTD (Nanchang Jiangxi) for micro-CT detection. Once scanning was accomplished, the region of interest (0.9 mm thickness) was chosen and utilized in 3D reconstruction. The analysis of data was carried out utilizing the ABA special bone analysis software, Magics 19.01, and Mimics 19.0 software. The following parameters were utilized for bone analysis: trabecular number (Tb. N), connectivity density (Conn. Dn), bone volume per tissue volume (BV/TV), trabecular thickness (Tb. Th), trabecular separation (Tb. Sp), cortical bone thickness (Ct. Th), and bone surface area (BS).

### Histological Analysis

For histopathological analysis, femurs samples that had been fixed were decalcified for 28 days utilizing 12 percent ethylenediaminetetraacetic acid (EDTA; pH 7.4). Subsequently, they were embedded in paraffin to conduct hematoxylin-eosin (H&E) staining or examine TRAP activity. The uSCOPE MXII Digital Microscope Slide Scanner (Microscopes International, Lubbock, TX, United States) was utilized to capture the images of the sections, which were then subjected to analysis on the Bioquent Osteo software (BIOQUENT, California, United States).

### Statistical Analyses

In the present research, we conducted all experiments a minimum of 3 times. The measurement units for the findings presented in the present research are in the form of mean ± standard deviation (SD). Statistical significances were on the basis of analysis of variance (ANOVA) or Student’s t-tests that were performed on the GraphPad Prism software version 8.0 (GraphPad Software, San Diego, CA, United States). Statistical significance was determined when the *p*-value recorded was less than 0.05.

## Results

### KEGG Enrichment Analysis of Iso Targets Related to Osteoporotic Disease

**FIGURE 1:**
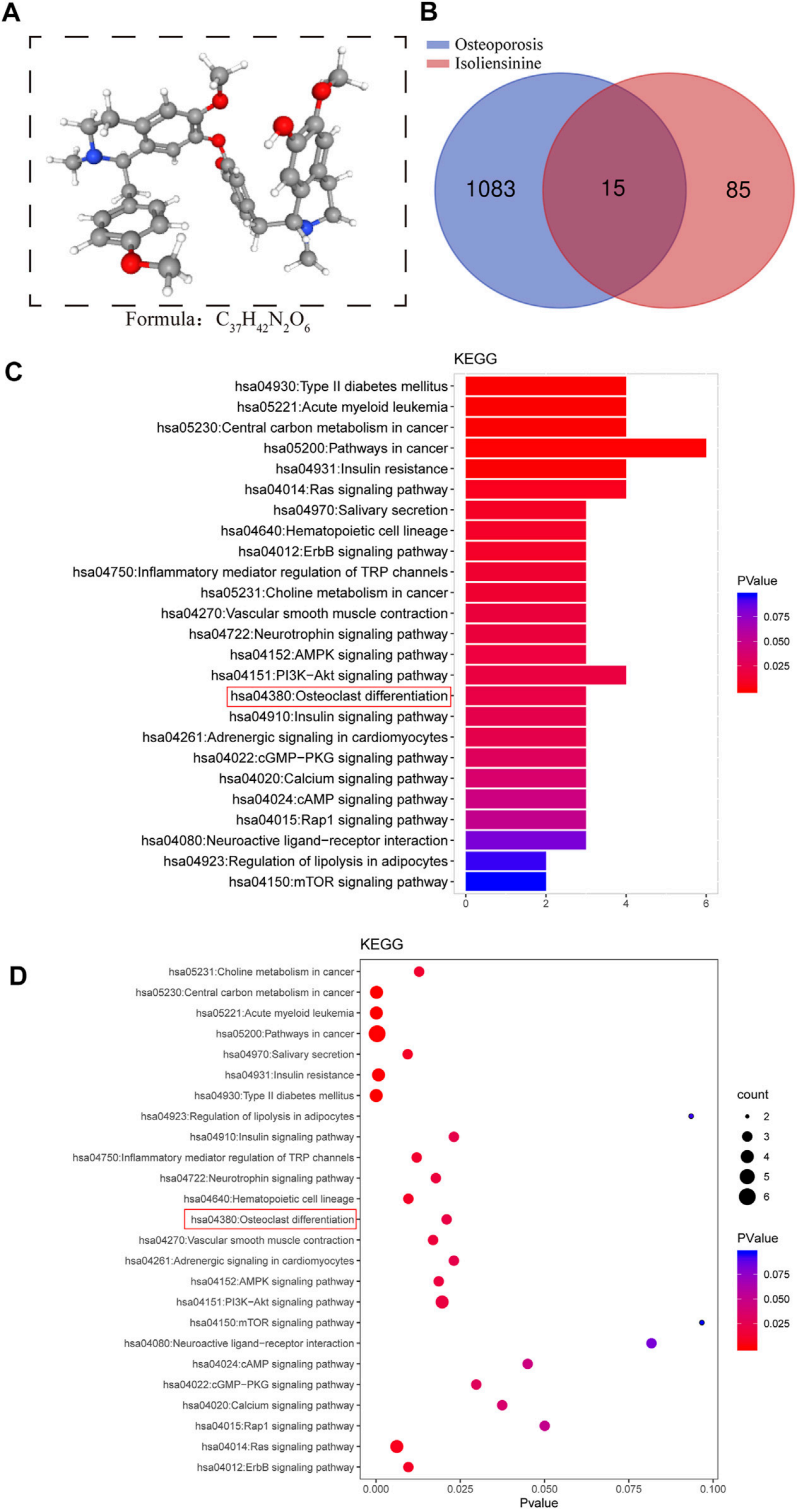
Search for targets of Iso related to osteoporotic disease through network pharmacology. **(A)** The 3D chemical structure and formula of Iso **(B)** Venn diagram of Iso prediction targets and targets related to osteoporotic disease **(C,D)** KEGG enrichment analysis of Iso target genes related to osteoporotic disease. The higher the correlation, the darker the color, the larger the enrichment index, and the larger the bubble.

We retrieved the 3D structural formula of the Iso drug through the PubChem database (Figure 1A), after obtaining the structural formula, we logged onto the website (http://www.swisstargetprediction.ch/) to Predict Iso target points and obtained 85 related targets. Then we logged on to the website (https://www.disgenet.org/search) to search for targets and obtained 1083 targets related to osteoporotic disease. After that, the predicted targets were imported into the Venny 2.1.0 platform together to make the Venn intersection graph (Figure 1B). ClusterProfiler (version 3.14.3) was used for GO and KEGG analysis, and we found that Iso mainly plays a role in osteoclast differentiation (Figures 1C,D).

### Iso Suppresses RANKL-Associated Osteoclastogenesis *in vitro*

**FIGURE 2:**
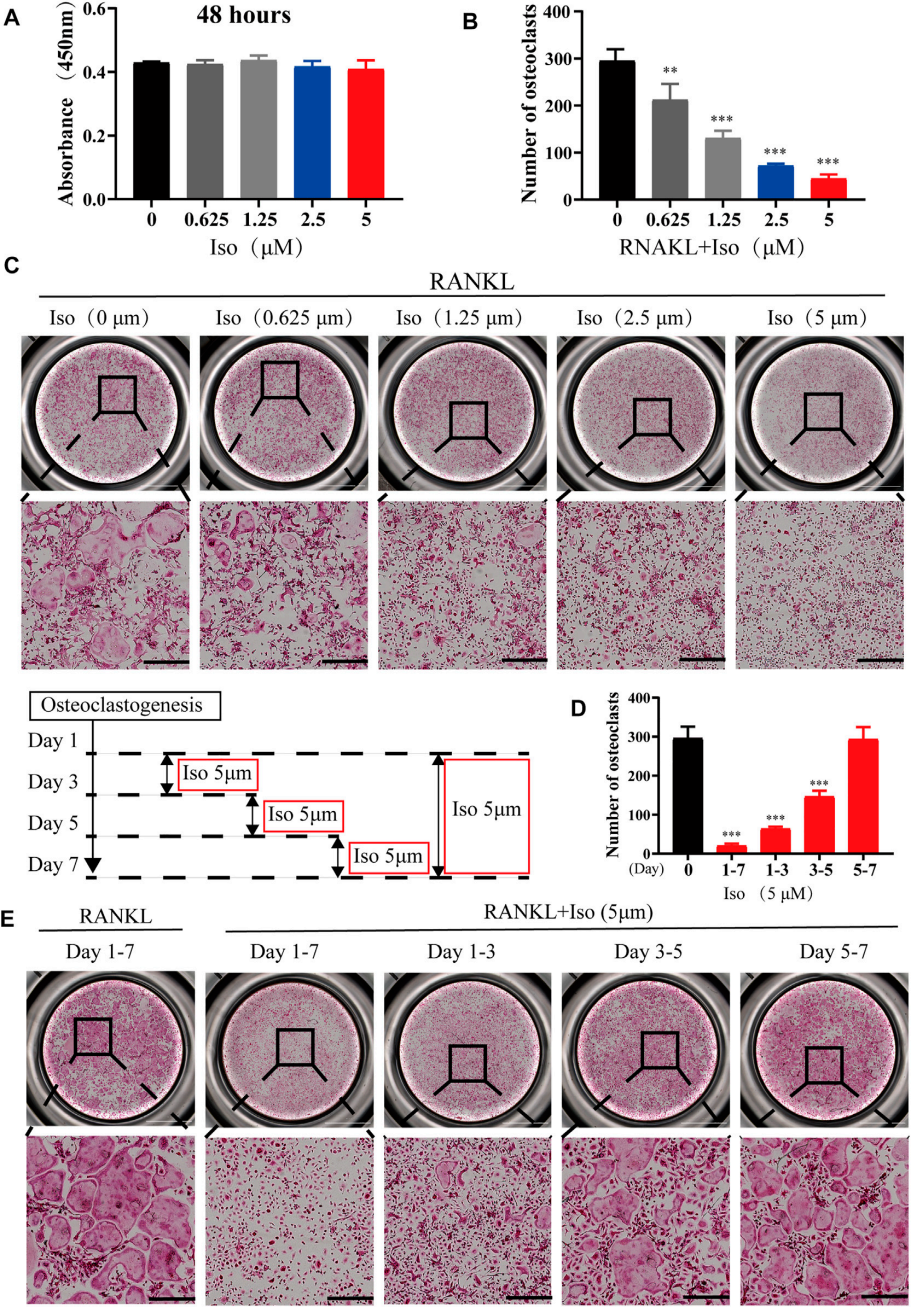
Iso suppresses RANKL-associated osteoclastogenesis *in vitro*. **(A)** The cellular cytotoxicity of Iso was evaluated by CCK-8 assay after 48 h of incubation **(B)** Quantification of TRAP-positive multinucleated cells (nuclei >3) (*n* = 3 per group). **(C)** Representative images of TRAP staining showing that Iso inhibited osteoclastogenesis dose-dependently. BMMs were stimulated with RANKL for 5 days in the absence or presence of indicated concentrations of Iso (scale bar = 500 μm). **(D)** Quantification of TRAP-positive multinucleated cells treated with Iso in different time periods (*n* = 3 per group). **(E)** Representative TRAP stained phase-contrast images of the time-dependent effect Iso on osteoclast formation. BMMs stimulated with RANKL and treated with 5 μM Iso on the indicated days were fixed and stained for TRAP activity (scale bar = 500 μm). The above data are expressed as the mean ± SD; *n* = 3; **p < 0.01 and ***p < 0.001. Iso: Isoliensinine; CCK-8: cell counting kit-8; BMMs: bone marrow macrophages; TRAP: tartrate-resistant acid phosphatase; RANKL: receptor activator of nuclear factor-κB ligand.

To rule out that Iso inhibits osteoclastogenesis is due to its own cytotoxicity, we tested the cytotoxicity of Iso at varying dosages (0, 0.625, 1.25, 2.5 and 5 μM) on BMMS for 48h by CCK8 assay. The results showed that there was no toxic effect on BMMs within the concentration range of 0.625 µM–5 µM (Figure 2A). Therefore, the maximal concentration of 5 μM was chosen for additional experiments. To thoroughly examine how Iso affects the differentiation of osteoclast, BMMs cells were used for the induction of osteoclast differentiation under the stimulation of M-CSF and RANKL while adding varying dosages of Iso (0, 0.625, 1.25, 2.5, and 5 µM). TRAP staining was performed after 6 days of induction (Figure 2C). The results showed that Iso lowered the number of osteoclasts in a dosage-dependent manner, and this effect was most obvious at 5 μM. Analysis of TRAP-positive osteoclasts with ≥3 nuclei and four Iso concentrations (0.625, 1.25, 2.5, and 5 μM) showed a substantial difference in the count of osteoclastic like cells (Figure 2B).

**FIGURE 3:**
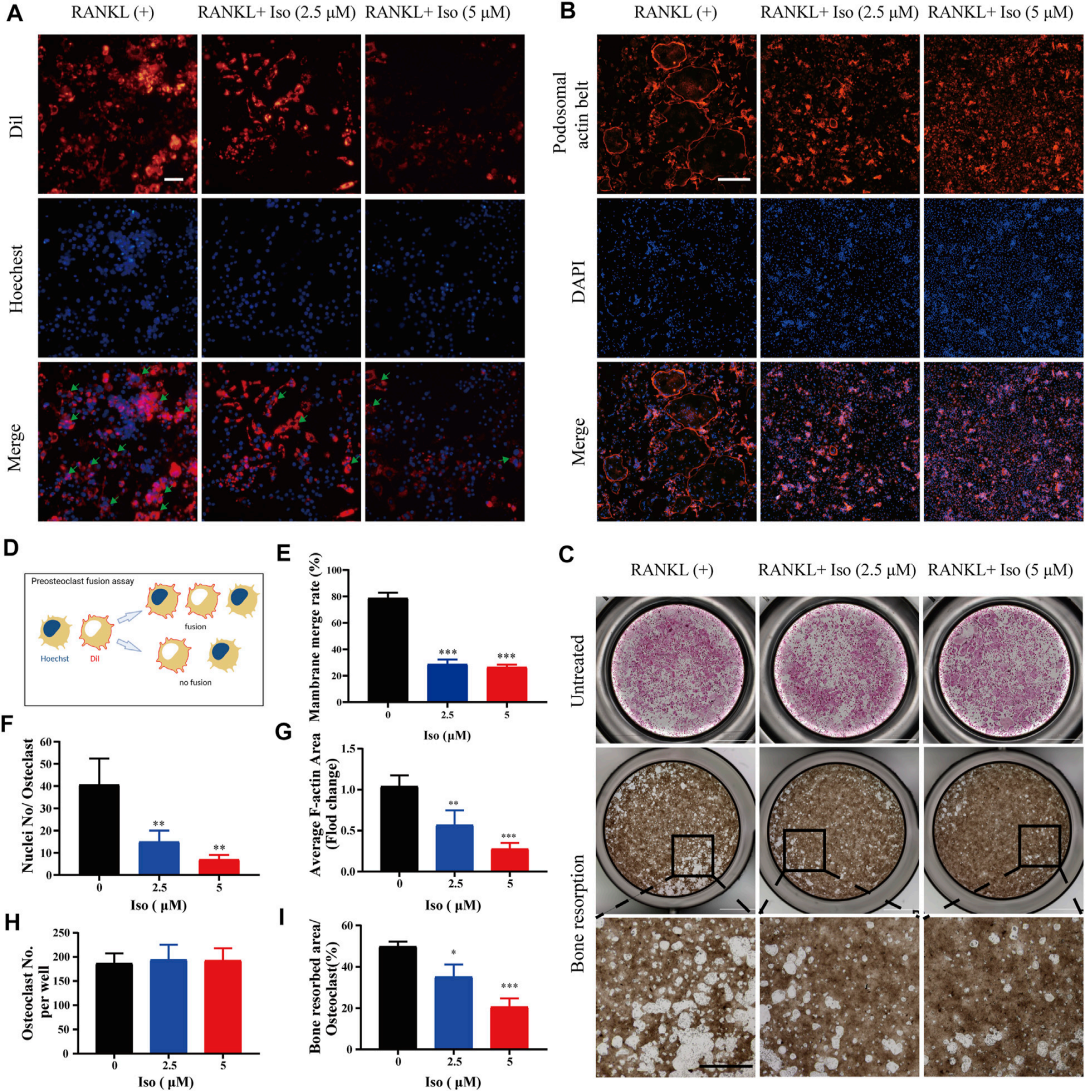
Iso affects osteoclast fusion, F-actin belt formation and attenuates osteoclastic hydroxyapatite resorption triggered by RANKL. **(A)** Representative images of osteoclast fusion treated with different concentrations of Iso (2.5 μM and 5 μM). The cell membrane (red) and nucleus (blue) of osteoclasts were stained (scale = 100 μM). **(B) **Representative images of podosomal belt formation in osteoclasts treated with different concentrations of Iso (2.5 μM and 5 μM). The actin cytoskeleton (red) and nucleus (blue) of osteoclasts were stained (scale = 400 μM). **(C)** Representative micrographs of TRAP-stained osteoclasts (upper panels) and the assay surface after removal of osteoclasts on hydroxyapatite-coated plates with or without Iso (2.5 μM and 5 μM) (scale = 1000 μM). **(D) **Process flows of osteoclast fusion assay **(E) **Double-fluorescence BMMs fusion on day 3 quantified by the membrane merge rate **(F)** Numbers of nuclei per osteoclast (*n* = 3). **(G)** Mean F-actin belt areas (*n* = 3). **(H,I)** The TRAP positive cells and resorbed area per well were quantitatively counted with ImageJ (*n* = 3). *p < 0.05, **p < 0.01, ***p < 0.001. All data are expressed as mean ± SD. TRAP: tartrate-resistant acid phosphatase.

To clearly clarify the time when Iso exerts its anti-osteoclast effect, treatment of the cells was performed using 5 μM Iso on Day 1, 3, and 5, and the cells were stimulated with RANKL. The positive control for the experiment comprised of the cells that did not receive treatment with Iso or were stimulated by RANKL. We discovered that Iso primarily suppressed the differentiation of osteoclast during the early and middle phases (day 1–5), particularly in the early phase (day 1–3) rather than in the later phase (day 5–7). Overall, this difference was statistically significant (Figures 2D,E). The osteoclast fusion assay further demonstrated that Iso inhibited the fusion of osteoclast precursors in the early phase, and in turn suppressed the formation of mature osteoclasts (Figures 3A,D). In addition, through one-way analysis of variance in contrast with the RANKL group, the difference is statistically significant (Figure 3E).

### Iso has Impacts on the Formation of the Podosome Belt in Osteoclasts and Suppresses Osteoclastic Hydroxyapatite Resorption

The F-actin ring is a specific cell trademark of mature osteoclasts, which is critical for the resorption function of osteoclasts. In our study, with varying dosages of Iso (0, 2.5, and 5 μM) intervention, the number of F-actin loops was significantly reduced, and it appeared a concentration gradient dependent (Figure 3B). In addition, through one-way analysis of variance in contrast with the RANKL group, Iso may considerably inhibit the average cell nucleus number of Osteoclasts and F-actin belt areas, and the difference is statistically significant (Figures 3F,G). The areas of F-actin belts were considerably lowered by Iso, implying that precursor cell fusion had been inhibited.

Next, we used the hydroxyapatite resorption method to detect the effect of Iso on the function of osteoclasts. Osteoclasts were seeded on hydroxyapatite-coated plates, followed by incubation in osteoclast culture medium containing RANKL under the intervention of varying dosages of Iso (2.5 μM and 5 μM). Once 48 h of incubation had elapsed, compared with the control group, the number of osteoclasts per well after 2.5 µM Iso and 5 µM Iso treatments did not change, while the resorption area of hydroxyapatite was significantly reduced (Figures 3C,H,I).

### Iso Inhibits Osteoclast Marker Gene Expression

**FIGURE 4:**
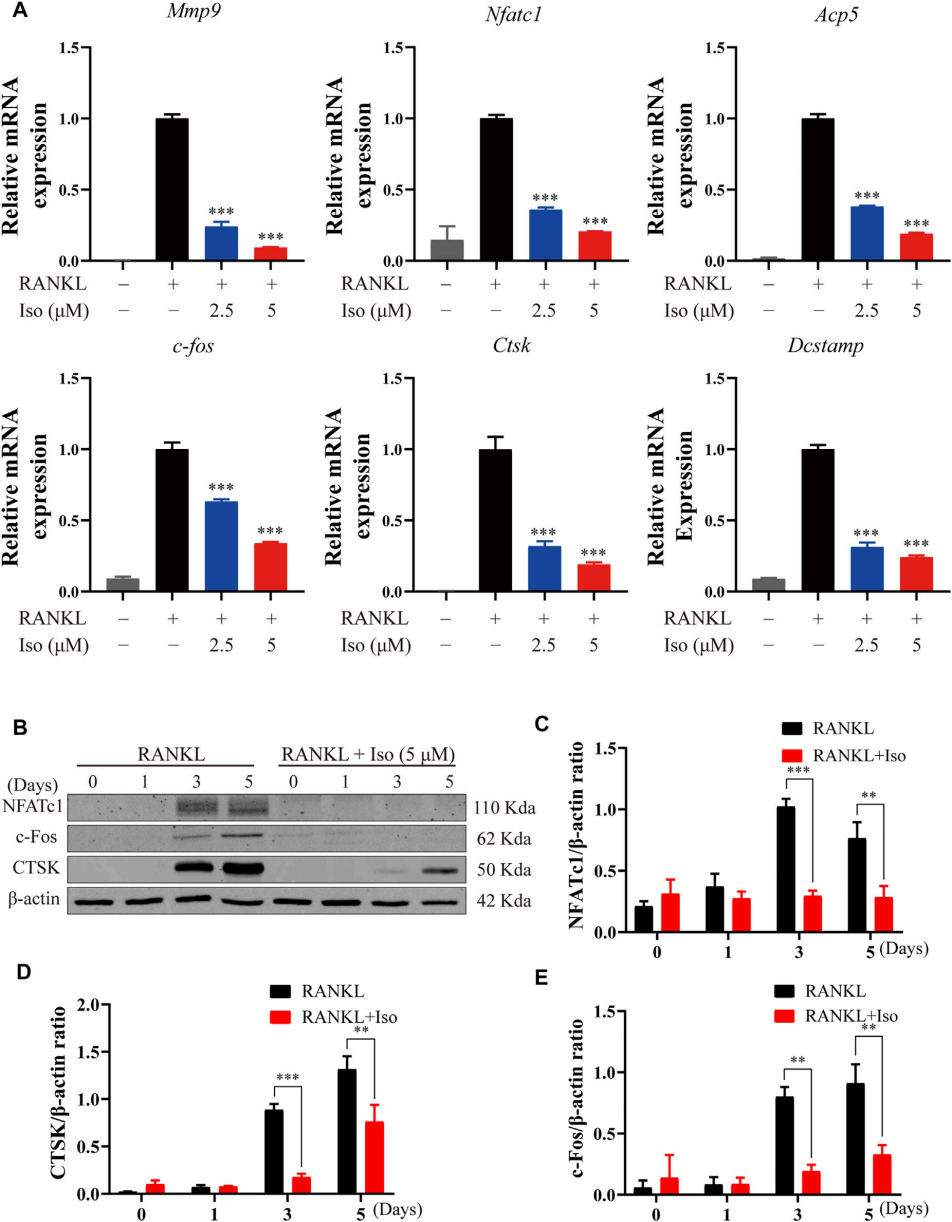
Iso inhibits osteoclast marker gene expression and attenuates c-fos, CTSK and NFATc1 protein expression. **(A)** BMMs were treated with M-CSF (25 ng/ml) and RANKL (50 ng/ml) in the presence or absence of the indicated concentrations of Iso. Gene expression was normalized to Hmbs. qPCR analysis of osteoclast-specific genes expression of *Mmp9, Nfatc1, Trap, C-fos, Ctsk* And *Dc-stamp* (*n* = 3 per group). **(B)** BMMs were treated with or without Iso (5 μM) on day1, day3, day5 and the proteins including *c-Fos, NFATc1* and *CTSK* were measured using western-blot **(C–E)** The expression of proteins mentioned above was quantitatively analyzed related to β-actin. *p < 0.05, **p < 0.01, ***p < 0.001. BMMs, bone marrow macrophages; Iso, Isoliensinine; PCR, polymerase chain reaction; RANKL, receptor activator of nuclear factor-κB ligand; M-CSF: macrophage colony stimulating factor; *Mmp9*, matrix metallopeptidase 9; *Nfatc1*, nuclear factor of activated T cells 1; *Trap*, tartrate-resistant acid phosphatase; *c-fos*, Proto-oncogene C-Fos; *Ctsk*, cathepsin K; *Dc-stamp*, dendritic cell-specific transmembrane protein.

To thoroughly study the inhibitory impact of Iso on osteoclastogenesis and bone resorption, we delve into if Iso reduces the mRNA expression of genes involved in osteoclast maturation. In this study, Iso (2.5 μM and 5 μM) inhibits the *Nfatc1* expression in a dosage-dependent way (Figure 4A). Additionally, Iso down-regulated expression of bone resorption–related genes, such as *Mmp9*, *Ctsk*, *Dcstamp*, *C-fos*, and *Apc5* (Figure 4A). These findings strongly indicated that Iso inhibited the osteoclast-specific genes expression and, as a consequence, osteoclastogenesis *in vitro.*

### Iso Abrogates RANKL-Associated NFATc1, c-Fos, and Ctsk Expression

The NF-κB activation induces c-Fos and NFATc1, which are known to be the main transcription factors for terminal osteoclasts differentiation. As a specific transcription factor, NFATc1 modulates the differentiation and maturation of osteoclasts. Loss of NFATc1 can lead to the complete loss of osteoclast bone resorption [3]. In line with the findings of the qPCR study, we discovered that Iso decreased the levels of protein expression of Ctsk, NFATc1, and c-fos (Figures 4B–E).

### Iso Reduces RANKL-Mediated Activation of NF-κB Signaling Pathways

**FIGURE 5:**
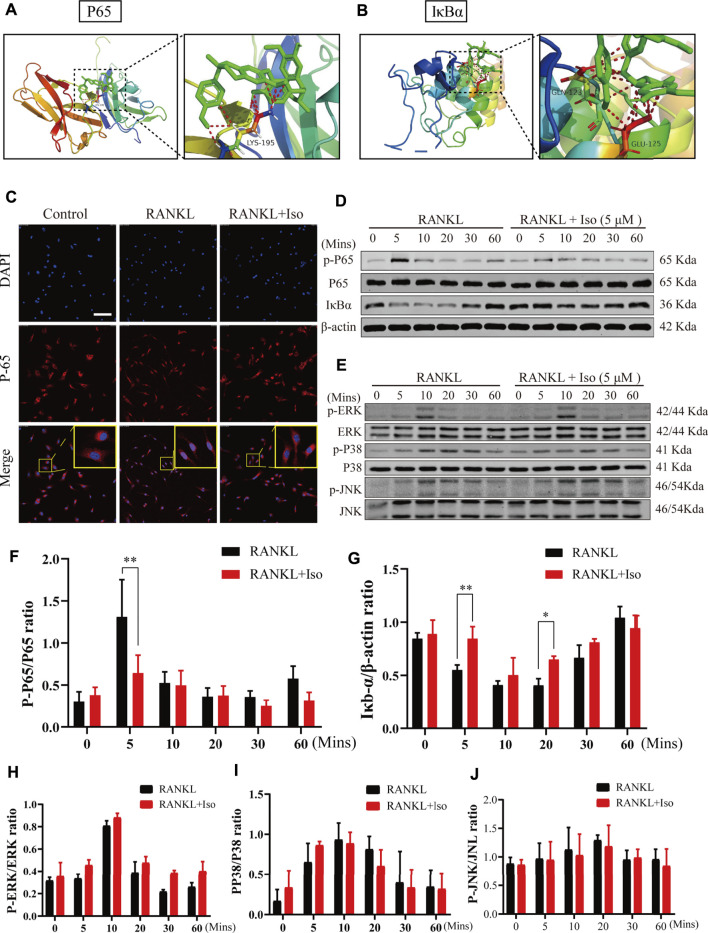
Iso abolishes RANKL-induced activation of NF-κB signaling pathways, but does not suppress MAPK pathway during osteoclastogenesis. **(A,B)** Molecular docking showed that Iso could bind with P65 and IκBα in NF-κB pathway **(C)** Immunofluorescence images of p65 nuclear translocation following RANKL stimulation without or with 5 μM Iso treatment (Magnification = ×20, scale = 100 μM). Cell nuclei were counterstained with DAPI. **(D,E)** Representative western-blot images of protein expression and phosphorylation status of p65, IκBα, ERK, JNK and p38 **(F–G)** The expression of proteins above was quantitatively analyzed standardized to β-actin **(H–J)** The expression levels of P38, ERK, JNK. All data are expressed as mean ± SD. *p < 0.05, **p < 0.01, ***p < 0.001.

Next, to explore the molecular mechanism of Iso in inhibiting osteoclasts, we first used network pharmacology to predict the possible target of Iso in osteoporosis. Bioinformatics analysis showed that Iso was mainly enriched in the osteoclast differentiation pathway and was closely related to the NF-κB pathway, which is consistent with previous research results (Shu et al., 2015; Zhang et al., 2015). According to our docking results, we found that Iso can bind to P38, JNK, ERK targets in MAPK pathway and P65, IκBα targets in NF-κB pathway. Compared with MAPK pathway, Iso could interact with the subunits of NF-κB pathways to form more stable conformation with a high binding energy of hydrogen-bond (Figures 5A,B; Supplementary Figure S1). Thus, we hypothesized that Iso performs a key function in inhibiting the differentiation of osteoclast via binding with the NF-κB proteins. Through the above experiments, we have confirmed that Iso can inhibit osteoclast differentiation and bone resorption. In the present research, we thoroughly examined the molecular processes of Iso inhibiting osteoclast production and function. As we all know, MAPK and NF-κB pathways are considered to be the primary signaling pathways that undergo activation in the process of osteoclastogenesis (Boyle et al., 2003). To examine the possible impact of Iso on the MAPK and NF-κB signaling pathways as well as the development of osteoclasts, we used Western blot to explore the effects of Iso on these two pathways.

NF-κB transcription factors bind to IκB-α and are maintained in a dormant state; nevertheless, these transcription factors become reactivated and released once IκB-α is degraded after being stimulated by RANKL. To thoroughly examine the NF-κB temporal inhibition by Iso, we evaluated the p-p65 and IκB-α expression at 0 min, 10 min, 20 min, 30 min, and 60 min following stimulation with RANKL. Our experimental results revealed that Iso inhibited the p65 phosphorylation at 5 min following stimulation with RANKL and inhibits the degradation of IkBα at 5 min (Figures 5D,F,G). At the same time, the confocal immunofluorescence experiment also showed that Iso can obviously prevent the nuclear translocation of p65 in osteoclast precursor cells (Figure 5C). It is consistent with the result that Iso mainly inhibits osteoclast differentiation in the early stage (day 1–3) in Figure 2E. In comparison, the results of our Western blot experiments show that Iso does not affect the expression of the main proteins p38, JNK, and ERK in the MAPK pathway (Figures 5E,H–J).

### Iso Attenuates OVX-Induced Bone Loss *in vivo*

**FIGURE 6:**
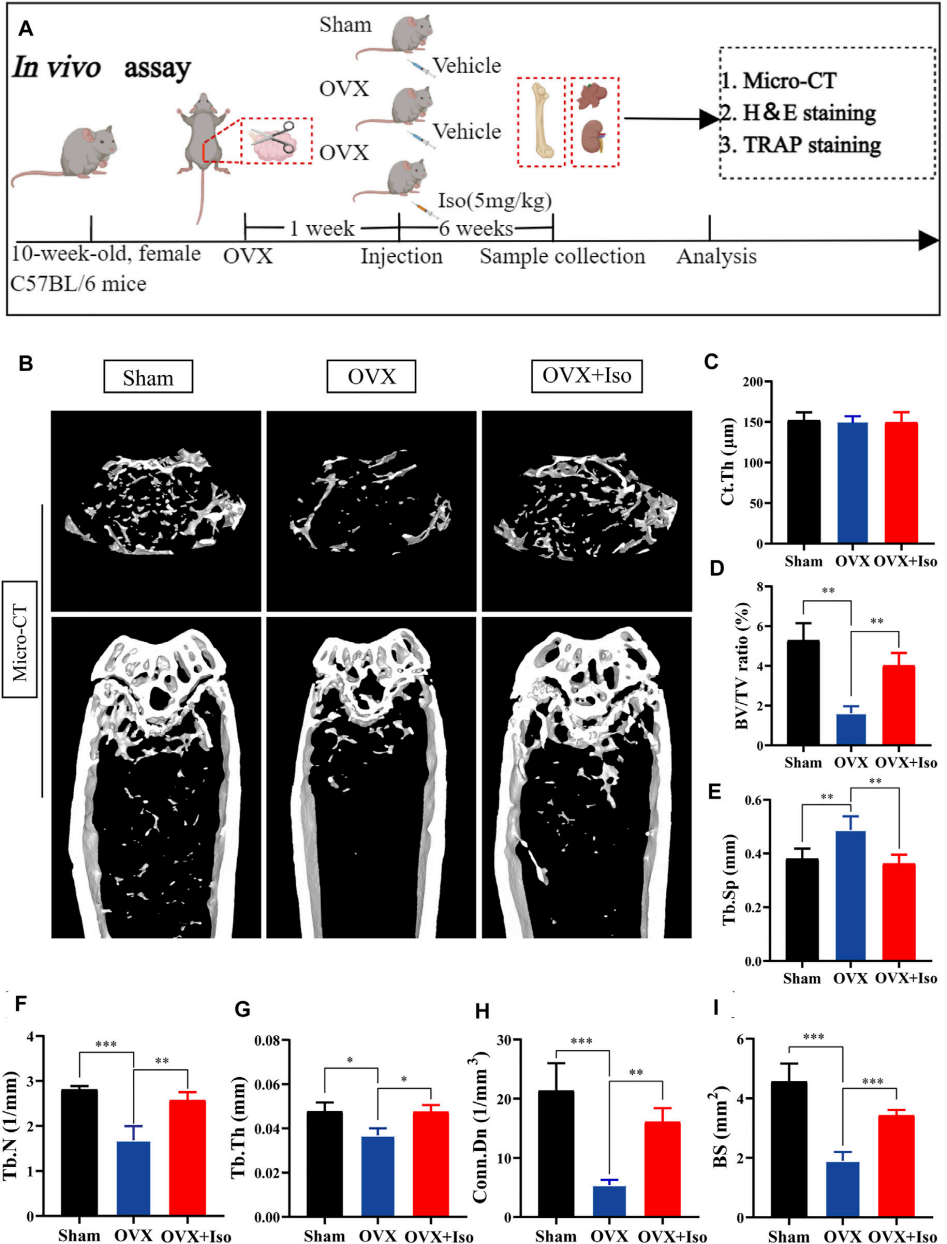
Iso attenuates bone loss in ovariectomized (OVX) mice. **(A)** Establishment of an osteoporosis model of OVX mice and an experimental design to evaluate the efficacy of Iso **(B–I)** Quantitative analysis of bone-related parameters, including Ct. Th, BV/TV Tb. Sp, Tb.N, Tb.Th, Conn. Dn, and BS (*n* = 5). The above data are expressed as the mean ± SD; *p < 0.05,**p < 0.01, and ***p < 0.001. Iso: Isoliensinine; Vehicle, 1% DMSO in PBS; TRAP, tartrate-resistant acid phosphatase. Ct. Th, cortical bone thickness; BV/TV, bone volume per tissue volume; Tb. Sp, trabecular spacing; Tb.N, trabecular number; Tb.Th, trabecular thickness; Conn. Dn, connectivity density; BS, bone surface.

Our findings confirm that Iso may inhibit the osteoclasts development and function *in vitro*. In order to further examine the inhibitory function of Iso *in vivo*, we used a mouse model of ovariectomy-stimulated osteoporosis. Mice receiving OVX or sham treatment were subjected to intraperitoneal injection with either Iso at a dosage of 5 mg/kg after every 2 days, or vehicle (PBS diluted in 1 percent DMSO) for a duration of 6 weeks following the surgical procedure (Figure 6A). The results of the HE staining revealed that Iso did not exhibit any significant harmful impact on the kidney or liver (Supplementary Figure S2). The findings of the micro-CT study demonstrated that Iso significantly attenuated bone mass loss in the distal femur in contrast with the OVX group (Figure 6B). Elevated Tb.Th, BS Conn. Dn, BV/TV, and Tb. N were discovered in the Iso group in comparison with the OVX mice that were not treated with Iso. Moreover, these findings were validated by a quantitative examination of bone characteristics. Tb. Sp, on the other hand, was reduced in the Iso groups, but the cortical bone metric Ct. th did not show any significant differences between the groups (Figures 6C–I).

**FIGURE 7:**
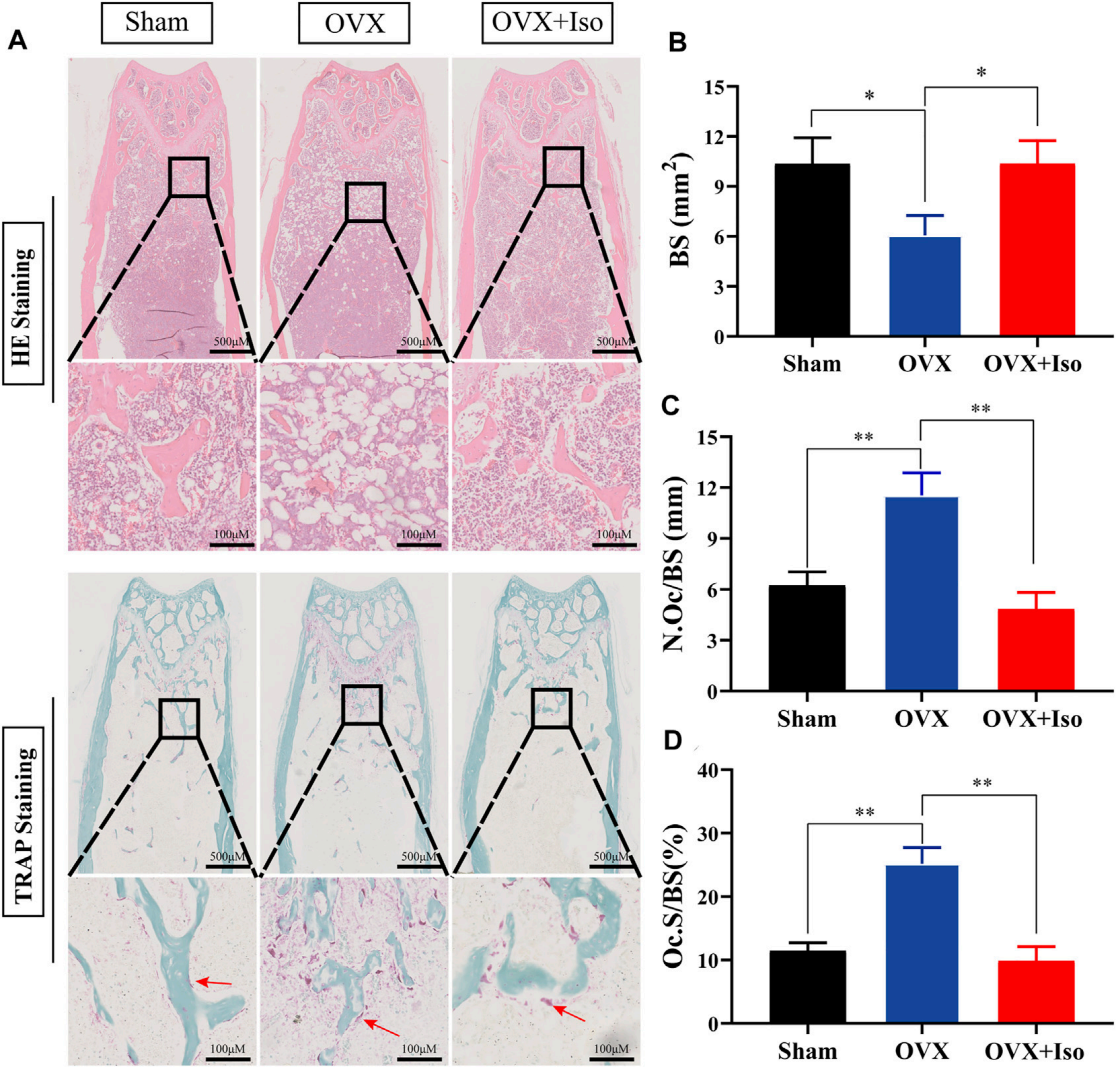
Iso treatment reduces the production of osteoclasts in ovariectomized mice. **(A)** Representative photographs of H&E and TRAP staining from each treatment group **(B–D)** Quantitative analysis of bone surface (BS), osteoclast number/bone surface (N.Oc/BS) and osteoclast surface/bone surface (Oc.S/BS). The data are expressed as the means ± SD; *p < 0.05, **p < 0.01, ***p < 0.001, *n* = 5. H&E, hematoxylin and eosin; TRAP, tartrate-resistant acid phosphatase.

The histological evaluation subsequently confirmed that OVX-induced bone loss was completely eliminated once the animals were treated with Iso every other day. The outcomes of the H&E staining indicated that the OVX-stimulated bone loss in mice was attenuated . Furthermore, TRAP staining indicated that the percentage of osteoclasts in the Iso therapy group dropped sharply (Figure 7A). After treatment with Iso, the number of osteoclasts per bone surface (N.Oc/BS), as well as the amount of osteoclast surface per bone (Oc.S/BS), were dramatically decreased; in contrast, the bone surface (BS) was enhanced (Figures 7B–D). Collectively, our findings illustrate that Iso has anti-bone resorption and anti-osteoclastic effects *in vitro* and has a protective impact on bone loss induced by OVX in mice *in vivo*.

## Discussion

Through network pharmacology, we have identified a potential target of Iso in osteoporosis. The results revealed that the target of Iso was concentrated in the osteoclast differentiation pathway, which was consistent with our previous osteoclast differentiation experiments, these results indicate that Iso may be a potential therapy for osteoporosis. We further found that Iso inhibited the differentiation as well as the maturation of osteoclasts in a dosage-dependent way *in vitro*, and the inhibition was most pronounced in the early stage (day 1–3) by suppressing the fusion of osteoclast precursors. In the late stage, osteoclast differentiation was almost not affected. Meanwhile, Iso significantly inhibited osteoclast-mediated bone resorption. And the results of the ovariectomized (OVX) animal model showed the same conclusion as well. Combining with the *in vitro* and *in vivo* experiments, we demonstrate that Iso might serve as a potential drug for osteoporosis.

To examine the molecular process that underlies the inhibitory impact of Iso on osteoclasts, we investigate the binding ability between Iso with the major protein molecules in NF-κB and MAPK pathway, which play a decisive role in osteoclast differentiation, by molecular docking analysis. Docking results pointed out that Iso possesses binding sites with NF-κB p65 in a high binding capacity.

The NF-κB pathway is the key pathway regulating osteoclast differentiation, which affects the differentiation of osteoclasts and bone resorption via regulating NFATc1 (Kim and Kim, 2016; Ono and Nakashima, 2018). Osteoclasts are distinctive cells for bone resorption and are derived from hematopoietic progenitor cells (McDonald et al., 2021). Osteoclast precursor cells showed and upregulated c-Fos and NFATc1 expression under RANKL stimulation, finally differentiated into mature osteoclasts (Grigoriadis et al., 1994; Ikeda et al., 2004). NF-κB activation is an essential step during the differentiation of osteoclasts as well as their maturation (Jimi and Ghosh, 2005). During the initial differentiation stages, RANK signals are mediated by recruiting tumor necrosis factor receptor-associated factor 6 (TRAF6) and the activation of NF-κB, AP-1, and MAPKs, (Park et al., 2017). The activation of NFATc1 induced by activated NF-κB is a critical modulator of osteoclast differentiation (Kim and Kim, 2016). NF-κB p65 is the key protein in the classical NF-κB pathway, so we further conduct the molecular docking analysis between Iso and NF-κB p65. The high molecular binding ability of Iso to NF-κB p65 protein suggested that Iso could inhibit osteoclast formation by binding to NF-κB p65 protein residues. Our osteoclast differentiation results strongly support the hypothesis.

Next, we investigated the molecular mechanism to reveal how Iso specifically regulates the NF-κB pathway. NF-κB plays an irreplaceable function during the differentiation of osteoclast. NF-κB is not active in the cytoplasm in a stable state, but it is translocated to the nucleus by classical or alternative pathways after being activated by RANKL (Ghosh and Karin, 2002). In the classical pathway, IKK complex (composed of IKK γ IKK *ß* and IKK α (commonly known as NF-κB Essential Regulator: Nemo)) phosphorylates IKK-κb to ubiquitinate and degrade it, thereby releasing NF-κB p65 into the nucleus. NF-κB dimer activation induces NFATc1 and c-Fos expression, thus stimulating mature osteoclasts to form and perform bone resorption functions (Ikeda and Takeshita, 2016). The phosphorylation of NF-κB p65 was attenuated by Iso at 5 min after RANKL stimulation via Western blot assay. Meanwhile, the confocal immunofluorescence assay also indicated that Iso suppressed the nuclear translocation of NF-κB p65 in osteoclast precursor cells. The results above were consistent with that Iso inhibited osteoclast differentiation mainly in the early stage (day 1–3). These results may be mediated by the inhibition of IκBα phosphorylation by Iso.

MAPK pathway regulates NFATc1-mediated osteoclast differentiation and bone resorption as well (Boyle et al., 2014). MAPK pathway induces osteoclast differentiation and maturation through protein kinase p38, JNK, and ERK (David et al., 2002; Böhm et al., 2009; Okamoto et al., 2017). Interestingly, the results of our experiment were not consistent with previous research (Zhang et al., 2015). Our Western blot results illustrated that Iso exhibited no significant impacts on the expression of p38, JNK, and ERK in the MAPK pathway. These results suggest that the effect of Iso on MAPK might be cell type dependent.

Activating NF-κB has been shown to induce the major transcription factors NFATc1 and c-Fos. NFATc1 is a specific transcription factor responsible for modulating the differentiation and maturation of osteoclasts. The deficiency of NFATc1 leads to complete disability of bone resorption (Takayanagi et al., 2002; Aliprantis et al., 2008). NFATc1 activates osteoclast-specific genes such as *Ctsk*, *Mmp-9*, *Acp5(Trap)*, and *Dcstamp* which stimulate mature osteoclast to form and perform bone resorption (Matsuo et al., 2004; Kim et al., 2008; Miyamoto et al., 2012). Dcstamp performs an indispensable function in the osteoclasts precursors fusion to generate polynuclear macrophages, and the formation of pre-polynuclear macrophages is a prerequisite for osteoclasts to perform bone resorption (Kukita et al., 2004; Yagi et al., 2005). MMP-9 and CTSK are involved in the breakdown of organic components in the bone matrix during bone resorption (Ono and Nakashima, 2018). Following western blot and RT-PCR analysis, we discovered that Iso considerably suppressed the expression of *Ctsk*, *Acp5(Trap)*, *c-fos*, Nfatc1, *Mmp-9* and *Dcstamp*. In conclusion, our results show that Iso mediates osteoclast inhibition by reducing downstream associated protein targets (such as NFATc1) via affecting the NF-κB pathway.

Iso is one kind of bibenzyl isoquinoline alkaloid with antioxidant, anti-inflammatory, and anti-cancer activities (Cheng et al., 2021). In this study, Iso dysregulates osteoclast differentiation by mediating the NF-κB pathway, not the MAPK pathway. *In vivo* validation of our ovariectomized mouse model also confirmed that Iso significantly reverses estrogen deficiency -induced bone loss. Our results deliver positive evidence on the promising preclinical application of Iso in preventing and treating osteolytic illnesses including osteoporosis.

**FIGURE 8:**
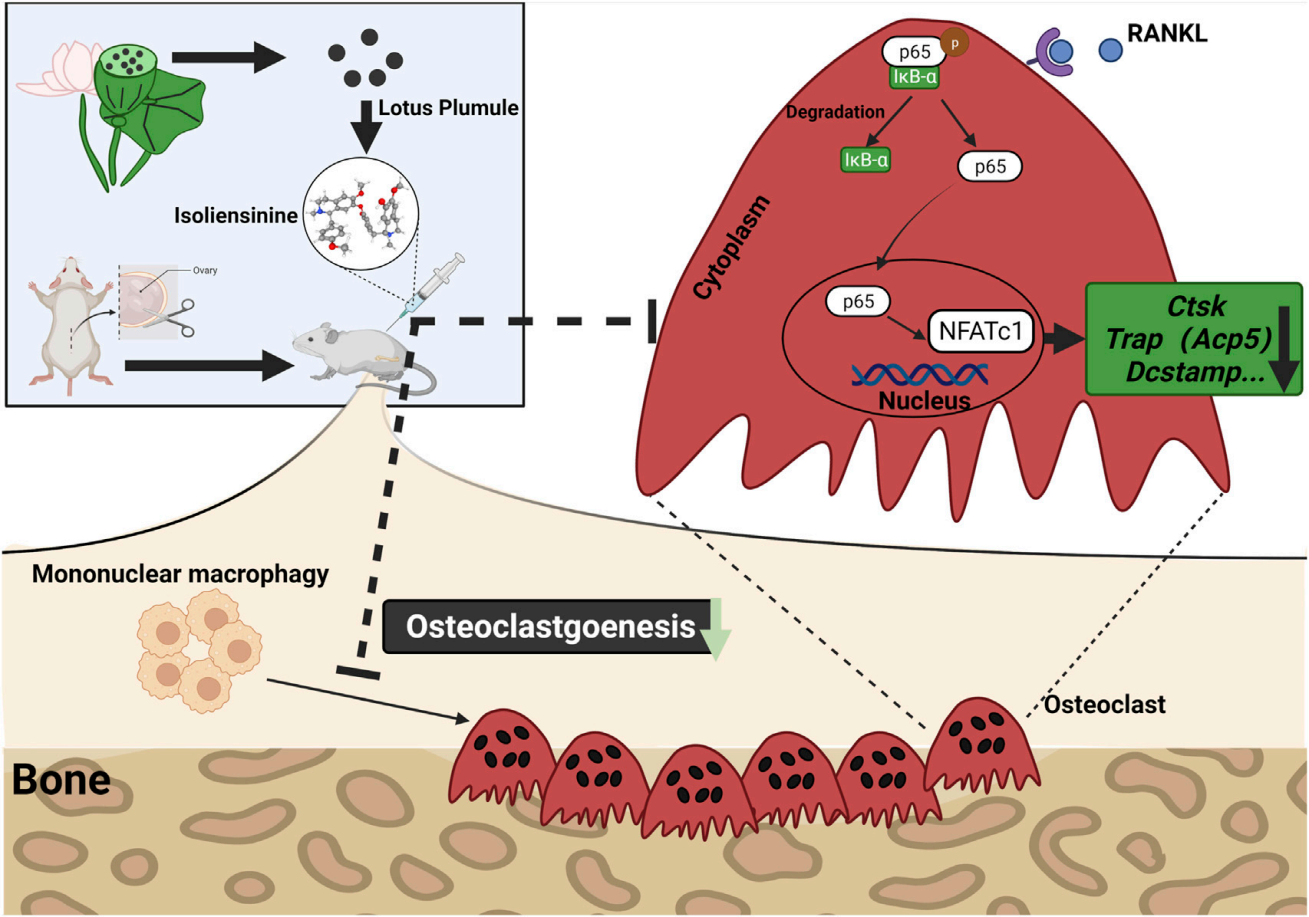
Iso treatment reduces the production of osteoclasts in ovariectomized mice. A proposed scheme for the inhibition of Iso on osteoclastogenesis. Upon RANKL binding to RANK, NF-κB pathways are activated, leading to the amplification of NFATc1. Several osteoclast-specific genes such as c-Fos, Ctsk, Trap, and Dc-stamp are upregulated as a result. RANKL: receptor activator of nuclear factor-κB ligand. NF-κB, nuclear factor-κB; NFATc1, nuclear factor of activated T cells 1, c-fos, Proto-oncogene, Ctsk, cathepsin K; Trap, tartrate-resistant acid phosphatase; Dc-stamp, dendritic cell-specific transmembrane protein.

In conclusion, our experimental data suggest that Iso suppresses the formation of osteoclasts and bone resorption induced by RANKL by mainly targeting NF-κB pathway. The molecular mechanism of the process of the differentiation of RANKL-induced osteoclasts *in vitro* showed that Iso could inhibit the IκBα degradation and NF-κB p65 nuclear translocation in osteoclast precursor cells, and then decrease the NFATc1 activation and the CTSK and c-Fos expression. Furthermore, intraperitoneal injection of Iso attenuates the bone loss induced by estrogen deficiency in OVX mice (Figure 8). In summary, Iso is a promising alkaloid compound as an osteoclast differentiation inhibitor in the treatment of osteolytic diseases.

## Data Availability

The datasets presented in this study can be found in online repositories. The names of the repository/repositories and accession number(s) can be found in the article/Supplementary Material.

## Supplementary Material

Supplementary Figure S1 Molecular docking of MAPK pathway Molecular docking showed that Iso could bind with P38, JNK and ERK targets in MAPK pathway

Supplementary Figure S2 Iso has no obvious toxic effect on the heart, liver or kidney in ovariectomized mice

## Abbreviations

c-fos, Proto-oncogene C-Fos; Mmp9, matrix metallopeptidase 9; Ctsk, cathepsin K; Acp5, acid phosphatase 5; Nfatc1, nuclear factor of activated T cells 1; Dc-stamp, dendritic cell-specific transmembrane protein; GAPDH: glyceraldehyde-3-phosphate dehydrogenase.
