# Supplementary figures and images for: Isoliensinine Suppresses Osteoclast Formation Through NF-κB Signaling Pathways and Relieves Ovariectomy-Induced Bone Loss
Source: Front Pharmacol. 2022 Jul 22;13:870553. doi: 10.3389/fphar.2022.870553 (PMC9353689; doi:10.3389/fphar.2022.870553)

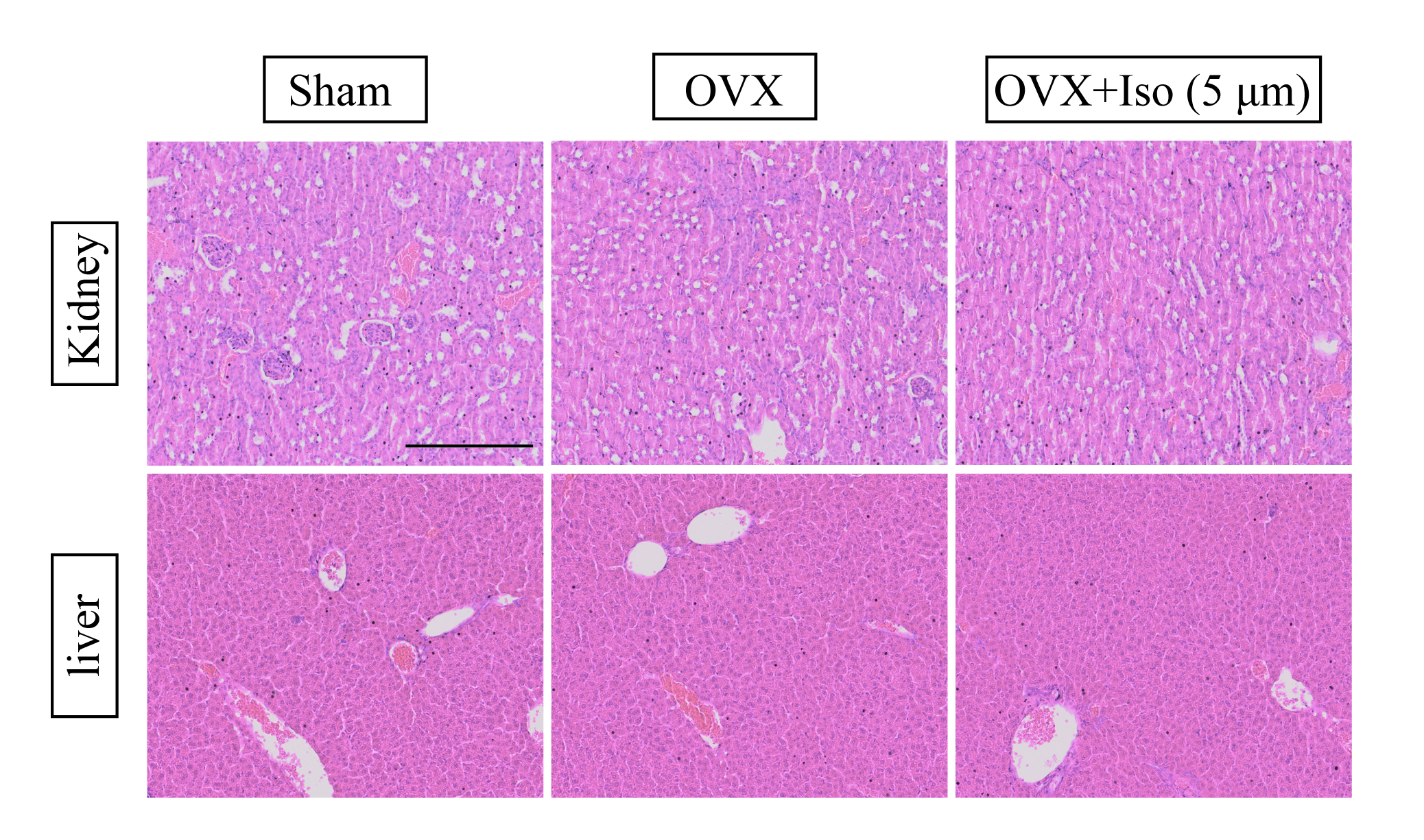

Supplement: Supplementary file 1 [file Image2.TIF]

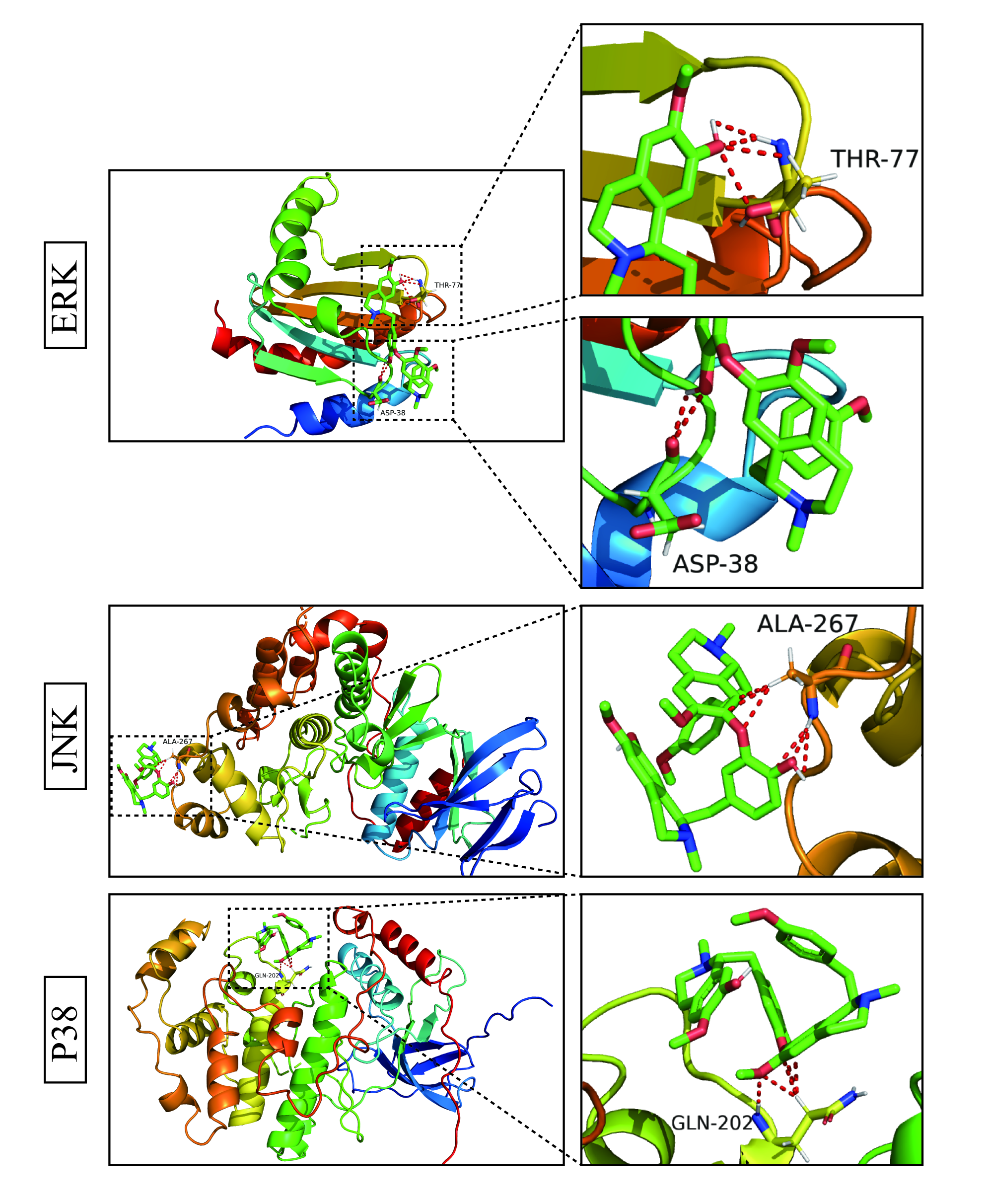

Supplement: Supplementary file 2 [file Image1.TIF]
